# Serum Acylated Ghrelin Is Negatively Correlated with the Insulin Resistance In the CODING study

**DOI:** 10.1371/journal.pone.0045657

**Published:** 2012-09-20

**Authors:** Peyvand Amini, Danny Wadden, Farrell Cahill, Edward Randell, Sudesh Vasdev, Xihua Chen, Wayne Gulliver, Weizhen Zhang, Hongwei Zhang, Yanqing Yi, Guang Sun

**Affiliations:** 1 Division of Medicine, Memorial University of Newfoundland, St. John’s, Newfoundland and Labrador, Canada; 2 Discipline of Laboratory Medicine, Memorial University of Newfoundland, St. John’s, Newfoundland and Labrador, Canada; 3 Division of BioMedical Sciences, Memorial University of Newfoundland, St. John’s, Newfoundland and Labrador, Canada; 4 Department of Physiology and Pathophysiology, School of Basic Medical Sciences, Peking University, Beijing, People’s Republic of China; Brigham & Women’s Hospital, Harvard Medical School, United States of America

## Abstract

**Objective:**

Ghrelin is a 28-amino acid orexigenic peptide synthesized mainly in the stomach. Acute administration of ghrelin has been found to decrease insulin secretion. However, little data is available regarding whether ghrelin contributes to the long-term regulation of insulin resistance at the population level. The aim of this study is to investigate the association between circulating ghrelin and insulin resistance in a large population based study.

**Design:**

A total of 2082 CODING study (Complex Diseases in the Newfoundland population: Environment and Genetics) subjects were assessed. Subjects were of at least third generation Newfoundland descent, between the ages of 20 and 79 years, and had no serious metabolic, cardiovascular, or endocrine diseases. Ghrelin was measured with an Enzyme Immunoassay method. Insulin and fasting glucose were measured by Immulite 2500 autoanalyzer and Lx20 clinical chemistry analyzer, respectively. Homeostatic Model Assessment of β cell function (HOMA-β) and Insulin Resistance (HOMA-IR) and Quantitative Insulin-sensitivity Check Index (QUICKI) were used for measurement of insulin resistance.

**Results:**

Partial correlation analyses showed a significant negative correlation between circulating ghrelin and insulin level and insulin resistance in the entire cohort and also in men and women separately. The aforementioned correlation was independent of age, percentage of trunk fat and HDL-cholesterol. According to menopausal status, only pre-menopausal women revealed negative correlations.

**Conclusion:**

Our results suggest that except for postmenopausal women, high circulating ghrelin level is associated with lower insulin resistance in the general population.

## Introduction

Diabetes mellitus is one of the most common chronic diseases worldwide. The estimated number of diabetic patients in 2010 was 366 million globally and it is predicted that this number will increase to 552 million by 2030 [1]. Type 2 diabetes is the most common type of diabetes. As a complex disease, both genetic and environmental factors are involved in development of this disease [2]. Pathogenesis of type 2 diabetes is complicated by several factors and insulin resistance, insulin deficiency, or both may contribute to this disease [3].

The gastrointestinal tract (gut) is the largest endocrine organ of the body. The gut produces hormones that have important roles in controlling body weight and energy homeostasis through the gut-brain axis [4], [5]. Previous studies showed that gastric bypass surgery results in a significant improvement of type 2 diabetes, and gut hormones play a role in this remission [6].

Ghrelin is a 28 amino acid orexigenic peptide synthesized mainly in stomach [7]. Circulating levels increase during fasting and decrease rapidly after a meal, so ghrelin has a role in acute changes in energy balance and satiety [8]. Ghrelin is a pleiotropic hormone that can influence different metabolic functions such as increasing food intake, inducing positive energy balance, promoting enlargement of adipocytes and also releasing growth hormone [9], [10]. Function of ghrelin on energy metabolism has been thought to be mediated by the central mechanisms, such as activation of the ghrelin receptor in the hypothalamic neuropeptide Y and agouti-related protein (NPY/AgRP) neurons [11]. Recently accumulating data suggest that ghrelin has central and peripheral effects on glucose regulation and insulin level [12], [13].

In rats ghrelin inhibits insulin secretion and stimulates glucagon secretion from pancreatic islets [14]. Infusion of exogenous ghrelin in healthy humans decreases glucose stimulated insulin secretion [12], [15], [16]. Moreover, it was shown that fasting ghrelin in type 2 diabetic patients is lower than in those who do not have diabetes [17]. Adolescent obese polycystic ovarian syndrome (which is characterized by insulin resistance) patients had lower ghrelin level compared with lean subjects and ghrelin was negatively correlated with Homeostatic Model Assessment of Insulin Resistance (HOMA-IR) [18].

On the other hand, no association was found between insulin sensitivity measured with euglycemic hyperinsulinemic clamp and ghrelin level in men [19]. In a prospective follow up study no significant difference was found between the ghrelin levels of subjects who had normal glucose tolerance and those who developed impaired fasting glucose, impaired glucose tolerance and type 2 diabetes mellitus [20].

Because of the controversy of the data regarding the effect of ghrelin on insulin resistance we designed the present study to investigate the association between ghrelin and insulin resistance in a large population based study: the CODING study (The Complex Diseases in the Newfoundland population: Environment and Genetics study).

## Methods

### Study Population

A total of 2082 subjects (1582 women and 500 men) were enrolled from the ongoing nutrigenomics CODING study [21]. Volunteers were 1) between the ages of 20 and 79 years old; 2) of at least third generation Newfoundland descent 3) without any serious metabolic, cardiovascular, or endocrine diseases and 4) females were not pregnant at the time of the study.

### Serum Measurement

Venous blood samples were drawn from all volunteers following a 12 hour fasting period. Blood was collected into tubes with EDTA for plasma preparation and serum separator tubes (SST) with clot activator. SST tubes were centrifuged at 3500 rpm for 10 min and EDTA tubes were centrifuged at 1300 g for 15 min to separate serum and plasma respectively. Plasma and serum samples were stored in a −80°C freezer.

Serum ghrelin was measured with an enzyme immunoassay method (Human Acylated Ghrelin Enzyme Immunoassay Kit of Spibio-bertin pharma) with the specifity of 100%, intra-assay coefficient of variation (CV) of 5.7% and inter-assay CV of 17%. Insulin and fasting glucose were measured by Immulite 2500 immunoassay analyzer and Lx20 clinical chemistry analyzer (Beckman Coulter Inc.CA, USA) respectively.

### Insulin Resistance Measurement

Although euglycemic hyperinsulinemic clamp is considered as the gold standard for measurement of insulin resistance, previous studies have shown that there is a high correlation between insulin resistance measured with HOMA and QUICKI and one that is measured by euglycemic clamp (R = 0.88, p<0.0001 and r = 0.69, p<0.05) [22], [23].

Homeostatic Model Assessment of β-cell function (HOMA-β) and Insulin resistance (HOMA-IR) were calculated from fasting glucose and insulin levels using the equations [22]:
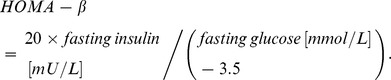


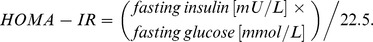



3 volunteers excluded from the study because of having fasting glucose lower than 3.5 mmol/L that result in negative values for HOMA-β.

Quantitative insulin-sensitivity check index (QUICKI) was the other insulin sensitivity index that was used for measurement of insulin sensitivity. It is determined by this mathematical equation [24]:
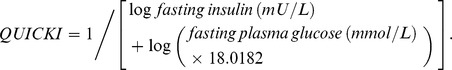



Subjects were divided into diabetic and non-diabetic groups (based on the history and glucose level). According to the 2006 WHO criteria fasting glucose 7.0 mmol/L was considered as diabetes [25]. 80 volunteers were in diabetic group and 2002 volunteers were in non-diabetic group.

### Anthropometric and Body Composition Measurements

Anthropometric measurements were taken with participants dressed in light clothing and without shoes. Standing height was measured to the nearest 0.1 cm using a stadiometer. Body weight was measured to the nearest 0.1 kg using a calibrated balance scale (Health O Meter, Bridgeview, IL). BMI was calculated from weight and height in kilograms per square meter. Waist circumference was measured midway between the lowest rib and iliac crest and hip circumference was measured at the widest point over the greater trochanters using a flexible tape measure and they were taken to the nearest millimeter.

Dual-energy X-Ray absorptiometry (DXA) Lunar Prodigy (GE Medical Systems, Madison, WI) was used for measurement of body composition.

### Medication Use and Menopausal Status

Volunteers were divided to medication users and non-medication users. Medication users were those who reported using prescribed medications or multivitamins regularly.

All of the female volunteers filled out a menstrual cycle and menopausal status questionnaire and they were categorized as premenopausal or postmenopausal based on this questionnaire.

### Statistical Analysis

SPSS version 18.0 was used for all of the statistical analyses. The summary statistics for continuous variables with normal distribution were expressed as mean and standard deviation. Non-normally distributed variables were expressed as median, minimum and maximum. The level of statistical significance was set at P value <0.05. Logarithmic transformation was used for the variables that did not have normal distribution (ghrelin, insulin, HOMA-IR, HOMA-β, QUICKI and Triglyceride). Analysis was performed on the entire cohort and also on men and women separately. Women were also divided into premenopausal and postmenopausal groups and the analysis was conducted within these two groups. Pearson correlation analyses were used to examine the relationship between various potential factors that may have an effect on ghrelin or insulin sensitivity. Partial correlation analyses (controlling for age, percentage of trunk fat and HDL-cholesterol ) were also performed. General linear model (multivariative analyses) was used to compare insulin resistance among the ghrelin groups, in which ghrelin groups were set by ghrelin tertiles and the covariates were age, trunk fat percentage and HDL-cholesterol. All analyses were repeated excluding diabetic patients for controlling the effect of blood glucose.

### Ethical Considerations

The current study was approved by the Human Investigation Committee of the Faculty of Medicine of Memorial University, St. John’s, Newfoundland, Canada and all of the volunteers signed informed consent to participate in the study.

## Results

### Physical and Biochemical Parameters

Mean and standard deviation of the parameters that were normally distributed are summarized in Table 1. Ghrelin, insulin, HOMA-IR, HOMA-β, QUICKI and Triglyceride were not normally distributed. Median and range of these values are shown in Table 2.

**Table 1:** Biochemical and body composition characteristics^1^.

	Entire Cohort	Women	Men
	(n = 2063–2085)	(n = 1571–1584)	(n = 492–501)
	Mean (SD)	Mean (SD)	Mean (SD)
**Age (y)**	42.92 (12.8)	43.75 (12.1)	40.29 (14.3)
**Weight (kg)**	73.64 (15.8)	69.93 (14.2)	85.41 (15.1)
**Height (cm)**	165.51(8.4)	162.26 (5.9)	175.81 (6.6)
**BMI (kg/m^2^)**	26.82 (5.1)	26.57 (5.2)	27.61(4.5)
**Waist (cm)**	92.13 (14.7)	90.49 (14.6)	97.36 (13.8)
**Hip (cm)**	101.23 (11.8)	101.65 (12.2)	99.90 (10.0)
**Body fat (%)**	35.01 (9.1)	37.95 (7.4)	25.66 (7.5)
**Trunk fat (%)**	37.23 (9.3)	39.30 (8.5)	30.66 (8.8)
**Android fat (%)**	42.54 (10.9)	44.27 (10.3)	37.03 (10.8)
**Gynoid fat (%)**	41.17 (9.6)	44.98 (6.4)	29.09 (7.8)
**Glucose (mmol/L)**	5.11 (0.8)	5.07 (0.8)	5.26 (0.8)
**Total Cholesterol(mmol/L)**	5.17(1.1)	5.21 (1.0)	5.04 (1.1)
**HDL-Cholesterol(mmol/L)**	1.46 (0.4)	1.54 (0.4)	1.21 (0.3)
**LDL-Cholesterol(mmol/L)**	3.14 (0.9)	3.12 (0.9)	3.18 (0.9)

1 All values are means ± Standard Deviations (SDs).

**Table 2:** Biochemical characteristics of data not normally distributed.

Variables	Entire Cohort		Female		Male	
	Median	Min-Max	Median	Min-Max	Median	Min-Max
**Ghrelin (ng/L)**	194.05	0.74–2339.95	193.44	0.74–2329.09	195.97	2.12–2339.95
**Insulin (pmol/L)**	55.90	14.40–272	55.55	14.40–272	56.95	14.40–270
**HOMA-IR** 1	1.78	0.41–16.01	1.77	0.41–16.01	1.87	0.41–10.92
**HOMA-β** 2	109.11	12.96–3801.3	111.16	12.96–3801.3	102.52	13.84–1382.29
**QUICKI** 3	0.35	0.26–0.45	0.35	0.26–0.45	0.35	0.27–0.45
**Triglyceride (mmol/L)**	1.01	0.23–5.88	0.98	0.23–5.88	1.16	0.31–5.54

1 HOMA-IR: Homeostatic Model Assessment of Insulin Resistance.

2 HOMA-β: Homeostatic Model Assessment of β cell function.

3 QUICKI: Quantitative Insulin-sensitivity Check Index.

### Association between Circulating Ghrelin Level and Insulin Resistance

Partial correlation analyses after controlling for confounding factors (age, percentage of trunk fat and HDL-cholesterol) showed negative correlation between circulating ghrelin level and insulin, HOMA-IR, HOMA-β and positive correlation between circulating ghrelin level and QUICKI. This association was not gender specific and it was reported in both male and female subjects (Table 3).

**Table 3:** Partial correlation of ghrelin with insulin resistance indexes after controlling for age, Percentage of trunk fat and HDL-cholesterol.

	Entire Cohort		Female		Male	
	r	P	r	P	r	P
**Glucose**	0.00	0.99	0.01	0.75	−0.03	0.52
**Insulin**	−0.09	0.00*	−0.07	0.00*	−0.11	0.02*
**HOMA-IR**	−0.08	0.00*	−0.06	0.01*	−0.10	0.02*
**HOMA-β**	−0.10	0.00*	−0.09	0.00*	−0.09	0.04*
**QUICKI**	0.08	0.00*	0.06	0.01*	0.10	0.02*

* P value <0.05.

### Influence of Menopause on the Relationship between Ghrelin and Insulin Resistance

To explore the influence of menopause on the association between ghrelin and insulin resistance, females were divided into pre-menopausal and post-menopausal groups and partial correlation analysis was performed after controlling for age, trunk fat percentage and HDL-cholesterol. In premenopausal women, there was a significant negative relationship between circulating ghrelin level and insulin, HOMA-IR, HOMA-β and positive correlation between circulating ghrelin and QUICKI whereas in post-menopausal women, there were no significant associations between ghrelin and insulin resistance factors (Table 4).

**Table 4:** Partial correlation analyses of ghrelin with physical and biomedical characteristics regarding menopausal status.

	pre-menopausal women		post-menopausal women	
	r	P	R	P
**Glucose (mmol/l)**	0.00	0.98	0.01	0.81
**Insulin (pmol/L)**	−0.08	0.02*	−0.06	0.14
**HOMA-IR**	−0.07	0.03*	−0.05	0.25
**HOMA-β**	−0.09	0.01*	−0.07	0.08
**QUICKI**	0.07	0.04*	0.05	0.26

* P value <0.05.

### Comparison of Insulin Resistance in Low, Medium and High Ghrelin Groups

**Figure 1:**
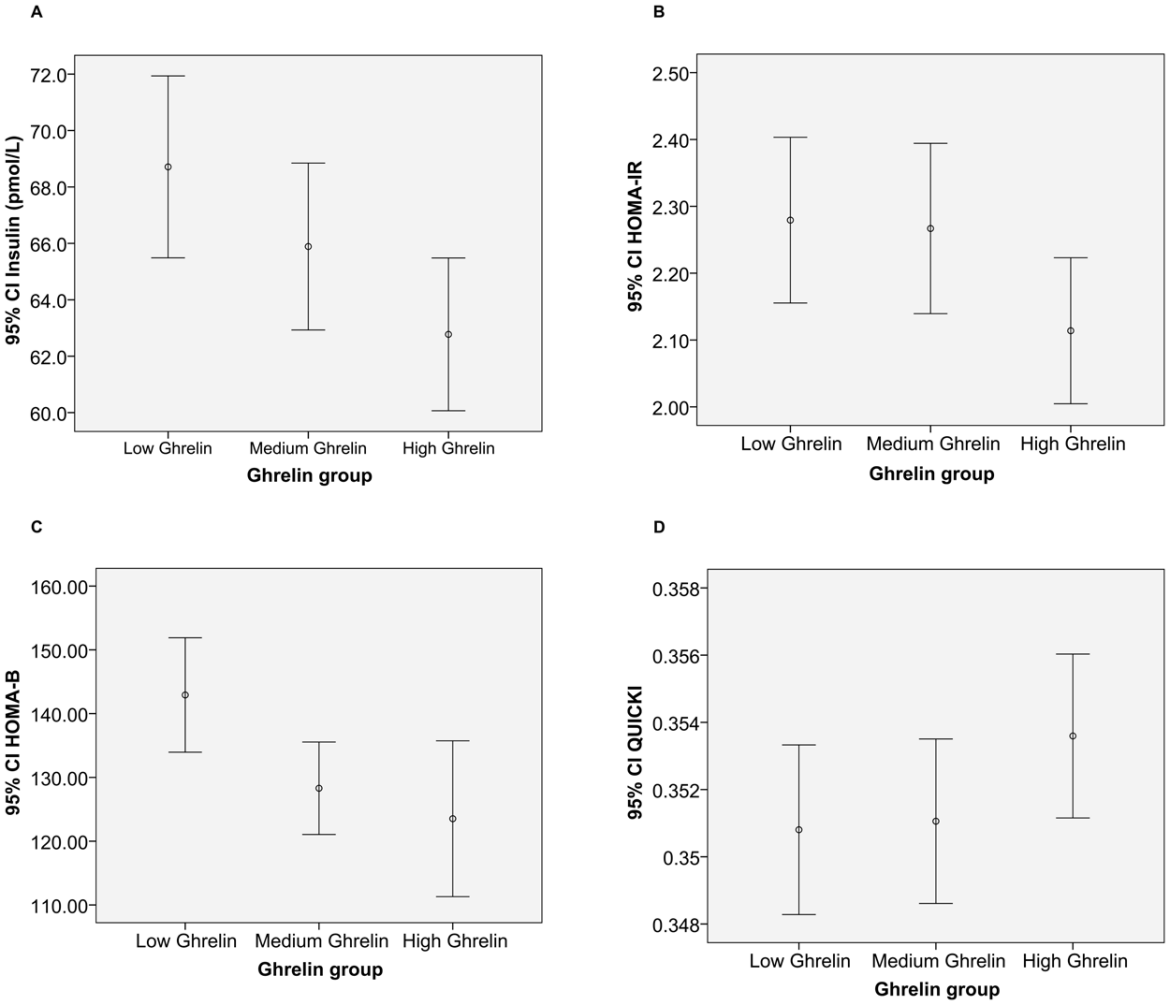
Error bars show the mean and 95% confidence interval for mean of insulin (A), HOMA-IR (B), HOMA-β (C) and QUICKI (D) in high, medium and low ghrelin groups.

After dividing volunteers in to three groups based on the ghrelin tertile, general linear model (multivariative analysis) was used for the comparison of insulin resistance between low, medium and high ghrelin groups. For insulin, HOMA-IR, HOMA- β and QUICKI there was no significant difference between low and medium ghrelin level or medium and high ghrelin levels but significant difference was evident between low and high ghrelin level (p value = 0.003, 0.01, 0.00 and 0.02 respectively). Figure 1 shows the mean and 95% confidence interval for mean of insulin, HOMA-IR, HOMA-β and QUICKI in high, medium and low ghrelin groups.

### Comparison of Ghrelin Level between Diabetic and Non-diabetic Subjects

Repeating the analysis after excluding the volunteers who report a history of diabetes and volunteers who had fasting blood glucose more than 7 mmol/L, no significant changes in the results were present.

### Association between Fasting Ghrelin and Age and HDL-cholesterol

Pearson correlation analyses showed no significant relationship between circulating ghrelin level and body composition characteristics, either in the total cohort or in the males and females separately. There was a positive correlation between fasting ghrelin level and age in the entire cohort (r = 0.08 and p = 0.00) and in females (r = 0.12 and p = 0.00) but not in males (r = −0.02, p = 0.72). No association was found between circulating ghrelin level and HDL-cholesterol.

## Discussion

The major finding in the present study is that circulating fasting ghrelin level is negatively correlated with insulin resistance and beta cell function in our CODING study. These findings were consistent using both HOMA and QUICKI as indices of insulin resistance. More importantly, our results indicate that the association of ghrelin with insulin resistance and secretion is independent of age, body composition and circulating HDL cholesterol. To our knowledge this is the largest study to evaluate the effect of ghrelin on insulin resistance, with the most comprehensive controls for major confounding factors, in the general population.

A number of experimental studies have evaluated the effect of ghrelin on insulin secretion in humans. Broglio et al. and Tong et al. showed that acute administration of ghrelin induced inhibitory effects on insulin secretion. These effects seem to be dose dependent and non-growth hormone mediated [12], [15]. In patients with metabolic syndrome, ghrelin was inversely correlated with insulin level and insulin resistance measured by HOMA-IR [26]. In a cross sectional study, low ghrelin level has been shown to be associated with type 2 diabetes and insulin resistance in middle-aged subjects and these associations remained significant after adjustment for sex, BMI and age [17]. Our findings provide further evidence that ghrelin level is negatively correlated with insulin level and resistance.

In pancreatic β-cells, ghrelin can inhibit glucose-induced insulin release via Gα_i2_-mediated activation of voltage dependent K^+^ channels and diminish action potential in β-cells [27]. Moreover, high ghrelin level may down-regulate growth hormone or its receptors and decrease insulin secretion secondarily [28]. Ghrelin receptors have been identified in the pancreas and ghrelin is produced partly in islet ε cells of the pancreas. Therefore the inhibitory effect of ghrelin on pancreatic β-cells might partly be due to paracrine mechanisms [15], [29]. Lower insulin resistance might be a compensatory response to decreased insulin level to maintain blood glucose within a normal range.

In contrast, there are some studies that did not report any association between ghrelin level and insulin resistance. A group in Sweden did not observe association between ghrelin level and insulin sensitivity measured by euglycemic hyperinsulinemic clamp in 104 subjects after adjustment for fat free mass [19]. Measurement of insulin resistance with euglycemic hyperinsulinemic clamp was certainly good. However, the sample size was very small compared with our sample size. On top of that the older age in this study could hinder the detecting of signals because of increased use of medications and other common chronic diseases. In a longitudinal study with 5.1 year follow up on 201 subjects, glucose tolerance and baseline fasting ghrelin level were measured. They found that fasting ghrelin levels failed to predict the development of glucose intolerance or type 2 diabetes [20]. The major concern for this study is that ghrelin is only one of the many factors affecting the development of insulin resistance and type 2 diabetes, so the possible effect of ghrelin on insulin resistance cannot be excluded.

Also, there are studies that found a positive association between ghrelin level and insulin resistance. Vestegard et al reported increased insulin resistance in 6 healthy men and eight hypopituitary men on stable replacement therapy with growth hormone and hydrocortisone after acute administration of ghrelin [16]. Similarly, acute administration of ghrelin in 10 patients who had the total gasterectomy and truncal vagotomy, reduced insulin mediated glucose disposal rate [30]. Because of the small sample size the results of these studies were only suggestive.

Our results, together with the results from others, indicate that there is a very wide range of ghrelin concentration in the population. The large standard deviation of circulating ghrelin would require a very large sample size to achieve the statistical power to detect the effect of ghrelin on insulin resistance and other phenotypes. This factor has to be considered since most of the previous studies seemed to be under power because of the small sample size which could lead to false positive or false negative results.

As the only known orexigenic gut hormone, the role of ghrelin in the development of human obesity is still unclear. Data from animal studies indicate that ghrelin induces the accumulation of adipose tissue [31]. However, cross sectional studies in humans reported negative correlation between ghrelin and adiposity [32]. In our current study we did not find significant association between fasting ghrelin levels and any adiposity phenotype. Detailed information will be available in another paper from our laboratory.

Effect of age on circulating ghrelin is still unclear. Paik and Thoshinai reported an inverse correlation between ghrelin and age [33], [34] whereas Cummings et al. reported a positive correlation between ghrelin level and age and they suggest that rising ghrelin levels could play a role in the effect of aging on increasing body fat [35]. In the present CODING study a positive correlation was observed between circulating ghrelin and age. As indicated in the analysis, the effect of age on ghrelin has been properly controlled in the present study.

In postmenopausal women, insulin resistance increases likely due to the reduced levels of sex hormones and physical activity and increased body fat [36], [37], [38]. Several studies explored the effect of menopause on ghrelin level. Purnell et al. reported that menopausal status or hormone replacement therapy do not have any effect on the ghrelin levels [39]; while Soni et al. found that estrogen hormone therapy can decrease ghrelin level and discontinuing it can increase ghrelin level in post-menopausal women [40]. In our study we did not see any significant difference in ghrelin level between premenopausal and postmenopausal women. However, we found a significant association between ghrelin and insulin resistance only in premenopausal women and not in postmenopausal women. The factors involved in the absence of relationship between circulating ghrelin and insulin sensitivity in postmenopausal women could be complicated by many factors including reduced level of sex hormones and possibly use of medications.

Previous studies have reported that ghrelin can bind to HDL particles in the blood [41] and HDL cholesterol level might have some effect on the measurement of ghrelin [39]. However, in our CODING study no significant association between circulating ghrelin and HDL-cholesterol was found.

To control the potential effect of diabetes status on the results of our study all analyses were repeated after volunteers who had blood glucose more than 7 mmol/L and reported to have diabetes were excluded. All findings remained significant.

In summary, the relationship between fasting ghrelin level and insulin resistance was systematically evaluated in the large CODING study with more than 2000 adult subjects from the Newfoundland population. To our knowledge this is the largest general population based study on the relationship between fasting ghrelin level and insulin resistance. Major confounding factors including age, gender, menopausal status and HDL-cholesterol level have been carefully analyzed and properly controlled. With the strong statistical power we provide reliable evidence that ghrelin may be a factor that help to reduce insulin resistance at the population level. This association is absent in the postmenopausal women. However, because of the nature and limitations of a cross sectional study, longitudinal study is warranted to fill the knowledge gap.
